# Dynamic changes in circulating tumor DNA assessed by shallow whole‐genome sequencing associate with clinical efficacy of checkpoint inhibitors in NSCLC

**DOI:** 10.1002/1878-0261.13409

**Published:** 2023-03-21

**Authors:** Caterina Carbonell, Joan Frigola, Nuria Pardo, Ana Callejo, Patricia Iranzo, Augusto Valdivia, Ilaria Priano, Susana Cedrés, Alex Martinez‐Marti, Alejandro Navarro, Laura Lenza, Mireia Soleda, Javier Gonzalo‐Ruiz, Ana Vivancos, Miriam Sansó, Enric Carcereny, Teresa Morán, Ramon Amat, Enriqueta Felip

**Affiliations:** ^1^ Thoracic Cancers Translational Genomics Unit Vall d'Hebron Institut d'Oncologia (VHIO) Barcelona Spain; ^2^ Clinical Research Department Vall d'Hebron Institut d'Oncologia (VHIO) Barcelona Spain; ^3^ Oncology Department Vall d'Hebron Barcelona Hospital Campus Spain; ^4^ Cancer Genomics Laboratory Vall d'Hebron Institut d'Oncologia (VHIO) Barcelona Spain; ^5^ Balearic Islands Health Research Institute (IdISBa) Palma de Mallorca Spain; ^6^ Medical Oncology Department, Catalan Institute of Oncology Badalona, Hospital Universitari Germans Trias i Pujol, Badalona Applied Research Group in Oncology Institut Germans Trias i Pujol Barcelona Spain; ^7^ Department of Medicine Universitat Autònoma de Barcelona Spain

**Keywords:** aneuploidy, cfDNA, immune checkpoint inhibitors, liquid biopsy, NSCLC, shallow whole‐genome sequencing

## Abstract

Immune checkpoint inhibitors (ICIs) targeting the PD‐1/PD‐L1 axis are the main therapeutic option for patients with advanced non‐small cell lung cancer (NSCLC) without a druggable oncogenic alteration. Nevertheless, only a portion of patients benefit from this type of treatment. Here, we assessed the value of shallow whole‐genome sequencing (sWGS) on plasma samples to monitor ICI benefit. We applied sWGS on cell‐free DNA (cfDNA) extracted from plasma samples of 45 patients with metastatic NSCLC treated with ICIs. Over 150 samples were obtained before ICI treatment initiation and at several time points throughout treatment. From sWGS data, we computed the tumor fraction (TFx) and somatic copy number alteration (SCNA) burden and associated them with ICI benefit and clinical features. TFx at baseline correlated with metastatic lesions at the bone and the liver, and high TFx (≥ 10%) associated with ICI benefit. Moreover, its assessment in on‐treatment samples was able to better predict clinical efficacy, regardless of the TFx levels at baseline. Finally, for a subset of patients for whom SCNA burden could be computed, increased burden correlated with diminished benefit following ICI treatment. Thus, our data indicate that the analysis of cfDNA by sWGS enables the monitoring of two potential biomarkers—TFx and SCNA burden—of ICI benefit in a cost‐effective manner, facilitating multiple serial‐sample analyses. Larger cohorts will be needed to establish its clinical potential.

## Graphical abstract

The authors applied shallow whole‐genome sequencing on cfDNA to study the association between tumor fraction (TFx) or somatic copy number alteration (SCNA) burden and immune checkpoint inhibitors (ICIs) efficacy in patients with metastatic NSCLC. Samples were sequenced before and during treatment: although high baseline TFx is associated with higher ICIs efficacy, its assessment in on‐treatment samples was able to better predict clinical efficacy, regardless of the TFx levels at baseline.

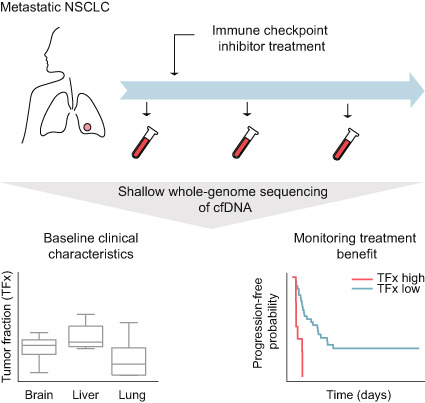

**Abbreviations**
ADK: lung adenocarcinoma
aTI: after treatment initiation
cfDNA: cell‐free DNA
ctDNA: circulating tumor DNA
DCB: durable clinical benefit
ddPCR: droplet digital PCR
ICIs: immune checkpoint inhibitors
NDB: no durable benefit
NSCLC: non‐small cell lung cancer
OS: overall survival
PD‐1: programmed cell death 1
PD‐L1: programmed death‐ligand 1
PFS: progression‐free survival
SCC: lung squamous cell carcinoma
SCNAs: somatic copy number alterations
sWGS: shallow whole‐genome sequencing
TFx: tumor fraction
TMB: tumor mutational burden

## 1 Introduction

Immune checkpoint inhibitors (ICIs) targeting the programmed cell death 1 (PD‐1)/programmed death‐ligand 1 (PD‐L1) axis have transformed the clinical management of patients with metastatic non‐small cell lung cancer (NSCLC) whose tumors bear no druggable oncogenic alteration. As a result, many patients are treated with this type of ICIs, alone, in combination with chemotherapy or in combination with other checkpoint inhibitors [1]. However, a substantial portion of patients do not benefit from this treatment, hence many studies have attempted to identify molecular and clinicopathologic biomarkers of benefit to these therapies [2, 3, 4, 5, 6, 7, 8]. PD‐L1 expression assessed by immunohistochemistry is used to select for pembrolizumab (an anti‐PD1 agent) monotherapy in first‐line [9, 10], although PD‐L1 remains a limited biomarker predicting ICIs efficacy [4, 11]. Tumor mutational burden (TMB) determined on tissue biopsy is positively associated with ICI benefit [2, 12, 13] and long‐term benefit in NSCLC [3]. Conversely, a high somatic copy number alteration (SCNA) burden has been associated with diminished benefit following ICI treatment in this disease [14]. Nevertheless, the availability of tumor tissue samples for comprehensive molecular testing in advanced NSCLC often can be limiting, particularly to assess disease evolution. To overcome this limitation, molecular profiling of analytes using bodily fluids—so‐called liquid biopsy—is emerging as a powerful tool, owing to its minimal invasiveness and serial testing capacity. Utilizing this technology to detect circulating tumor DNA (ctDNA) on cell‐free DNA (cfDNA) extracted from plasma has generated promising results. Few studies have found a positive association between baseline TMB, determined on cfDNA, and ICI benefit in advanced NSCLC [15, 16, 17].

Furthermore, several studies have indicated that an early on‐treatment reduction in the levels of the mutations detected on cfDNA is positively associated with ICI benefit [17, 18, 19, 20, 21]. Additionally, undetectable ctDNA at surveillance time points has been useful to identify and better stratify long‐term responders [22]. Nevertheless, these studies are based on relatively expensive gene‐panel sequencing or require previous knowledge of the genomic landscape of the tumor to follow one or several mutations [droplet digital PCR (ddPCR) or amplicon‐sequencing].

However, shallow whole‐genome sequencing (sWGS) on cfDNA is an inexpensive technique, does not require previous knowledge of specific alterations (unlike ddPCR), allows investigators to estimate the tumor fraction (TFx) and delivers a genome‐wide profile of SCNAs [23], thereby enabling estimation of the SCNA burden. Thus, to test the value of these two molecular features (TFx and SCNA burden) on cfDNA, we assembled a cohort of 45 patients with metastatic NSCLC treated with ICIs, extracted cfDNA from plasma samples obtained at different time points (before and after treatment initiation until disease progression) and applied sWGS. We then examined the association of these molecular features with the patient clinical profile (i.e., histology, metastatic lesions location) and treatment efficacy.

## 2 Materials and methods

### 2.1 Patient and non‐cancer donor information

Fifty‐six patients with metastatic NSCLC who were going to be treated with ICI‐based therapy (excluding combinations with chemotherapy) at Hospital Vall d'Hebron were prospectively enrolled between January 2017 and June 2019. Clinical information was retrieved revising electronic clinical information. Clinical data censoring was May 31^st^, 2022. Patients were treated with ICI‐based therapy administered as standard therapy or as part of a clinical trial. Responses to ICIs treatment were assessed using the Response Evaluation Criteria in Solid Tumors version 1.1 guidelines [24]. Metastatic lesions were recorded prior to ICIs treatment initiation.

Twelve donors without cancer gave blood for this study. Four were males and eight were females. Age was available for eight donors; median age was 54 years.

This study involves human participants and was approved by the ‘Comité de ética de investigación con medicamentos del Hospital Universitario Vall d'Hebron’ (PR(AG)308/2016). Participants gave written informed consent to participate in the study. The study methodologies conformed to the standards set by the Declaration of Helsinki.

### 2.2 Blood sample collection and plasma processing

Peripheral blood from patients and healthy donors was collected in BD Vacutainer EDTA Tubes (10 mL). For lung cancer patients, blood was collected at baseline, and approximately every cycle of treatment (2 or 3 weeks, depending on the protocol) until progressive disease.

Plasma was extracted within 2 h of blood collection employing two centrifugation steps of 10 min at 200 ***g*** and stored at −80 °C.

### 2.3 Extraction times selection

Samples were considered baseline (B) when drawn before ICI treatment initiation (up to 10 days prior to treatment), T_1_ when collected between 10 and 25 days after treatment initiation (aTI), T_2_ between days 30 and 50 aTI and T_3_ samples between days 60 and 100 aTI. Samples categorized as progression were obtained between 15 days before and 50 days after the date of radiological progression. Samples at T_1_, T_2_, and T_3_ were only considered if they preceded the date of progression.

### 2.4 cfDNA extraction

Plasma samples were thawed in a 37 °C water bath and centrifuged at 4 °C for 10 min at 16 000 ***g***. cfDNA was isolated from 1 to 2 mL of plasma using the QIAamp^®^ Circulating Nucleic Acid Kit (QIAGEN Strasse, Hilden, Germany) and quantified with a Qubit Fluorometer (ThermoFisher Scientific, Eugene, OE, USA).

### 2.5 sWGS sequencing

Between 4 and 20 ng of cfDNA was used for the preparation of barcoded libraries using the NEBNext^®^ Ultra™ DNA Library Prep Kit (New England Biolabs, Ipswich, MA, USA). For patients with less than 4 ng of cfDNA available, all cfDNA extracted from 1 to 2 mL of plasma was used for library preparation. Samples were subjected to low‐coverage whole‐genome sequencing on a HiSeq2500 platform to generate single‐end reads (50 bp) at a target mean coverage of 0.2×.

### 2.6 Shallow whole‐genome processing

#### 2.6.1 Sequence alignment

Sequencing reads were aligned to the GRCh38 reference genome using the mem algorithm of the bwa V.0.7.17 software. Duplicates were marked using the markduplicates tool from picard V.2.21.2.

#### 2.6.2 SCNA calling and processing

BAM files were transformed into WIG using hmmcopy's [25] readCounter with the window parameter set to 1 000 000 and the quality set to 20. Next, ichorcna [23] was used to call SCNAs, with the following parameters: ploidy: 2, 3, normal: 0.5, 0.6, 0.7, 0.8, 0.9, maxCN: 5, includeHOMD: False, chrTrain: 1 : 22, estimateNormal: True, estimatePloidy: True, estimateScPrevalence: True, scStates: 1, 3, txnE: 0.9999, txnStrength: 10 000, maxFracCNASubclone: 0.9, maxFracGenomeSubclone: 0.6, chrs: 1 : 22, chrTrain: 1 : 22. A custom panel of normals built from 12 blood samples served as a reference.

For each sample, the GC‐Map correction median absolute deviation (MAD) value was retrieved from the ‘.params.txt’ file generated by ichorcna. Those whose segmentation profiles had a MAD above 0.1 were discarded. ichorcna solutions were manually inspected to identify cases where a suboptimal solution had been chosen by the algorithm. When this happened, the apparently optimal solution was forced, as recommended in the software documentation.

An amplitude filter of ±0.05 was applied to the candidate SCNAs identified. Thus, we considered to be gained those regions identified as gained or amplified by ichorcna and whose amplitude was greater than 0.05, while we considered to be deleted those regions called as deleted by ichorcna and whose amplitude was lower than −0.05.

SCNA burden was computed as the sum of the number of base‐pairs affected by copy number alteration events encompassing whole arms and chromosomes as in Frigola et al. [14].

### 2.7 Progression‐free survival and overall survival computing

Progression‐free survival (PFS) was computed as the number of days between the ICI treatment initiation and the date of progression in those patients who presented a radiological progression. In those who did not, PFS was computed as the number of days between the ICI treatment initiation and the date of last follow‐up, where patients' PFS was censored.

Durable clinical benefit (DCB) was defined as remaining without disease progression for 6 months.

Overall survival (OS) was computed as the number of days between the ICI treatment initiation and the exitus date, when the event was considered to have occurred. For the remainder of the patients, the OS was computed as the number of days from the start of ICI treatment to the date of the patient's last follow‐up. The OS of these patients was censored at this date.

### 2.8 Survival models

Multivariate Cox proportional‐hazards models were built using the lifelines python library (10.5281/zenodo.4579431) with the step‐size parameter set to 0.5. Categorical variables were transformed into dummy variables, and numerical variables were standardized.

Kaplan–Meier curves were produced using the lifelines python library, and log‐rank tests to compare curves were performed using the lifelines.statistics logrank_test function.

### 2.9 Statistical analysis

Mann–Whitney Wilcoxon (MWW), Chi‐squared, Wilcoxon signed‐rank and Kruskal–Wallis tests were performed using the scipy python library [26].

## 3 Results

### 3.1 Patient cohort characteristics

Of the 56 patients prospectively enrolled, 45 patients had confirmed metastatic NSCLC, were treated with ICIs, had follow‐up and at least a baseline plasma sample, thus were included in the analysis. Thirty‐two patients (71.1%) were male, 43 (95.6%) were current or former smokers, and 32 (71.1%) had adenocarcinoma histology. Twenty‐one patients (46.7%) were treated in first‐line therapy and the rest in second. Patients were treated with different PD‐(L)1 agents alone or in combination with other checkpoint inhibitors. Median progression‐free survival (PFS) and overall survival (OS) were 4.6 (139 days) and 12.8 months (383 days), respectively. Detailed clinicopathologic characteristics can be found in Tables S1 and S2.

**Fig. 1:**
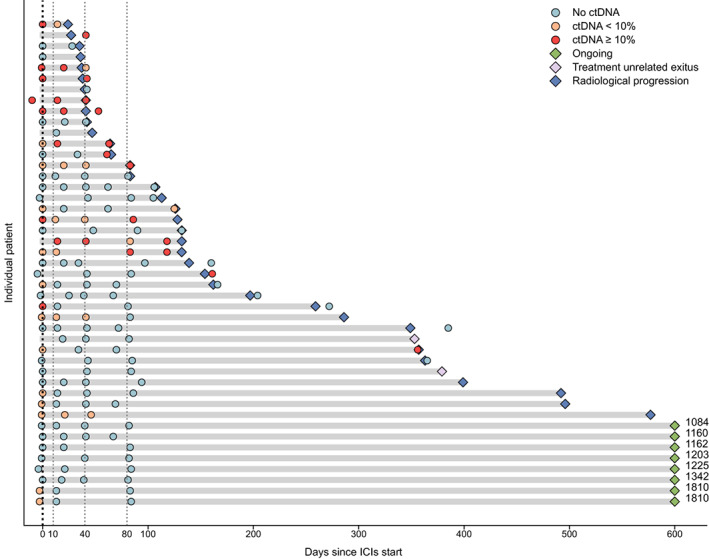
Swimmer plot showing the progression‐free survival time, vital status, time points assessed by sWGS and the corresponding value of Tumor Fraction. ctDNA, circulating tumor DNA; ICIs, immune check point inhibitors.

We retrospectively performed sWGS (average mean coverage: 0.30, SD: 0.15) on 173 samples, corresponding to 45 patients and 12 noncancer donors. Eight samples were discarded; two due to insufficient cfDNA quality and six because of elevated MAD (see methods). Thus, 12 noncancer and 153 tumor high‐quality samples were used in subsequent analysis. Among the tumor samples, 40 were taken prior to ICI treatment (referred to as baseline (B)) and 113 after treatment initiation (aTI). Specifically, 31 samples were taken at T_1_, corresponding to between 2 and 3 weeks aTI (median of 14 days aTI), 26 at T_2_ (median of 41 days aTI), 29 at T_3_ (median of 83 days aTI) and 27 near radiological progression (Fig. 1).

### 3.2 Clinical correlates of baseline cell‐free DNA tumor fraction

We detected ctDNA in 19 patients (47.5%) from whom a baseline (B) sample was available and passed the different quality controls (Fig. 1). Their median TFx was 9.2% and the maximum was 52.9%. Seven patients had a TFx ≥ 10%.

**Fig. 2:**
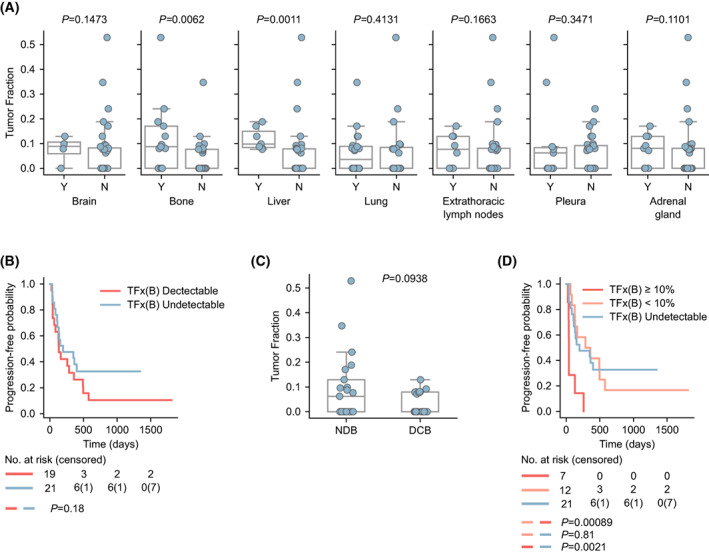
Clinical correlates of Baseline Tumor Fraction (A) Tumor fraction levels according to the presence (Y) or absence (N) of the indicated metastatic lesion. Mann–Whitney Wilcoxon, *P*‐values are shown. The boxes show the quartiles of the dataset, whereas the whiskers extend to show the rest of the distribution, except for points that are determined to be ‘outliers’ by the seaborn library. (B) Kaplan–Meier survival curves of progression‐free survival (PFS) in patients with undetectable ctDNA versus detectable ctDNA at baseline. Log rank test, *P*‐value is shown. (C) Tumor fraction according to no durable benefit (NDB) and durable clinical benefit (DCB). Mann–Whitney Wilcoxon, *P*‐value is shown. The boxes show the quartiles of the dataset, whereas the whiskers extend to show the rest of the distribution, except for points that are determined to be ‘outliers’ by the seaborn library. (D) Kaplan–Meier survival curves of progression‐free survival (PFS) in patients with undetectable ctDNA, patients whose Tumor Fraction (TFx) was < 10% and patients whose TFx was ≥ 10%. Log rank test, *P*‐values are shown.

First, we studied the relationship between TFx at baseline and different clinical features, such as histology, sex, smoking status, line of therapy, and none of them exhibited statistical significance (Fig. S1A). However, we found an association between certain metastatic lesions and TFx. Specifically, TFx was higher in patients presenting bone or hepatic metastatic lesions (Mann–Whitney Wilcoxon, *P* = 0.0062 and *P* = 0.0011, respectively, Fig. 2A).

Next, we examined the correlation between TFx at baseline and ICI efficacy. When considering two groups of patients, those with detectable ctDNA and those with undetectable ctDNA, there was a nonsignificant trend toward inferior PFS and overall survival (OS) in those patients with the former (Log rank test, *P* = 0.18, Fig. 2B; Log rank test, *P* = 0.22, Fig. S1B). When we compared patients with durable clinical benefit (DCB) to those with no durable benefit (NDB), TFx was higher in the latter group, although it did not reach statistical significance (Mann–Whitney Wilcoxon, *P* = 0.0938; Fig. 2C). To further study the value of TFx at baseline, we used a cut‐off of 10% as suggested by others [27, 28], which can be applied across different cohorts in contraposition to median or tertiles. Thus, we established the following three categories: undetectable ctDNA, detectable TFx < 10% and TFx ≥ 10%. When considering PFS as outcome, patients with a TFx ≥ 10% had shorter PFS than those patients with undetectable ctDNA or TFx < 10% (Log rank test, *P* = 0.0021 and *P* = 0.00089, respectively), whereas no differences were observed between the two latter groups (Log rank test, *P* = 0.81, Fig. 2D). Although it did not reach statistical significance, a similar trend was observed when using OS as the clinical endpoint (Fig. S1C, Table S3). Only one patient, who had a TFx ≥ 10% at baseline achieved DCB, whereas the rest of the patients with a TFx ≥ 10% (*n* = 6) did not achieve DCB (Fig. S1D). Importantly, after adjusting for several clinical parameters, TFx ≥ 10% retained a significant association with diminished PFS in a multivariate Cox regression model (*P* = 0.003, Table 1), also when incorporating the presence of metastatic lesions on different tissues (*P* = 0.0037, Table S4). A similar trend was observed when using OS as the clinical endpoint (*P* = 0.066, Table S5). For 30 patients, PD‐L1 status as a binary variable (positive vs. negative) was available, thus we adjusted for it in the multivariate analysis. In this model, TFx ≥ 10% remained significant (*P* = 0.04, Table S6).

**Table 1:** Multivariate Cox proportional‐hazards survival model of progression‐free survival at baseline.

Variable	HR	HR lower 95%	HR upper 95%	*P*‐value
Histology				
Other				
ADK	0.71	0.19	2.64	0.61
SCC	1.24	0.26	6.01	0.76
Smoking				
No				
Yes	0.79	0.18	3.54	0.76
Sex				
Male				
Female	2.42	0.95	6.18	0.065
ICIs treatment line				
First				
Second	4.08	1.52	10.93	0.0052
Tumor Fraction (TFx)				
TFx < 10%				
TFX ≥ 10%	4.44	1.66	11.88	0.0030

Altogether, our data indicate that, at baseline, high TFx associates with diminished ICI benefit when using a previously established threshold of 10%. Moreover, TFx associates with the presence of certain metastatic lesions, suggesting that it might provide useful prognostic information.

### 3.3 Value of on‐treatment determinations

Several studies, employing gene‐panel sequencing, have established the importance of ctDNA kinetics to predict therapeutic efficacy [17, 18, 19, 20]. We, therefore, applied sWGS on plasma samples drawn at different on‐treatment time points (Fig. 1). As specified above, we analyzed samples taken 2–3 weeks aTI (T_1_), around 40 days aTI (T_2_), and around 80 days aTI (T_3_).

**Fig. 3:**
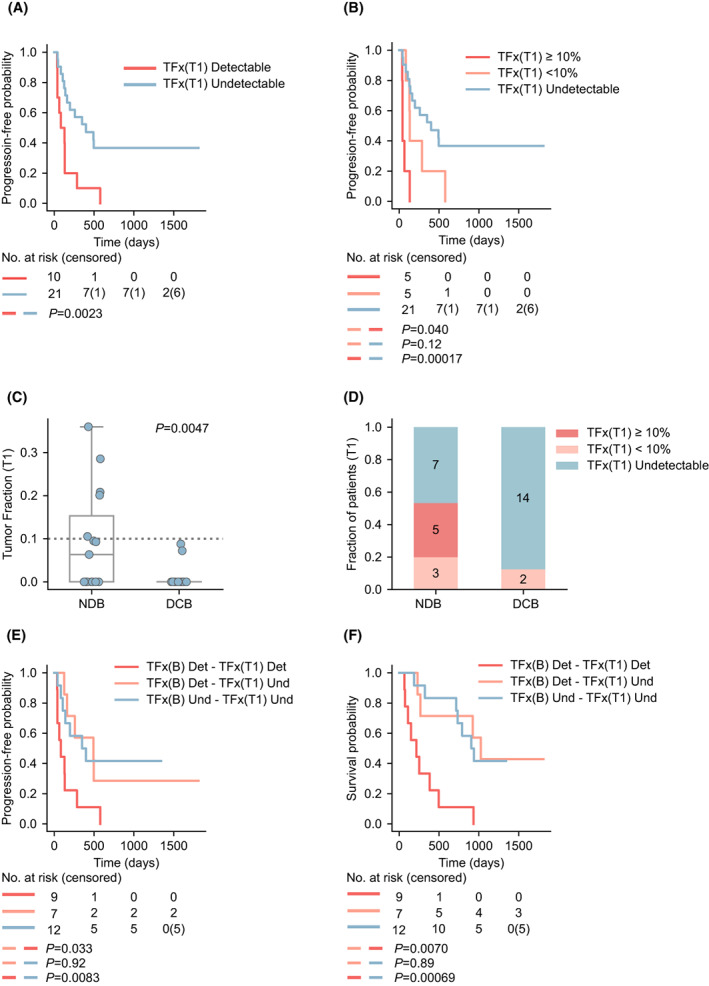
On‐treatment value of Tumor Fraction determination. (A) Kaplan–Meier survival curves of progression‐free survival (PFS) in patients with undetectable ctDNA versus detectable ctDNA at T_1_. Log rank test, *P*‐value is shown. (B) Kaplan–Meier survival curves of progression‐free survival (PFS) in patients with undetectable ctDNA, patients whose Tumor Fraction (TFx) was < 10% and patients whose TFx was ≥ 10% at T1. Log Rank test, *P*‐values are shown. (C) Tumor fraction according to no durable benefit (NDB) and durable clinical benefit (DCB) at T1. Mann–Whitney Wilcoxon, *P*‐value is shown. The boxes show the quartiles of the dataset, whereas the whiskers extend to show the rest of the distribution, except for points that are determined to be ‘outliers’ by the seaborn library. (D) Fraction of patients with Undetectable ctDNA, TFx < 10% and TFx ≥ 10% according to NDB and DCB. (E, F) Progression‐free survival (PFS) and overall survival (OS) in patients with undetectable ctDNA at baseline and undetectable ctDNA at T1 (TFx(B) Und ‐ TFx(T1) Und), detectable ctDNA at baseline and undetectable at T1 (TFx(B) Det ‐ TFx(T1) Und), or detectable ctDNA at both (TFx(B) Det ‐ TFx(T1) Det). Log rank test, *P*‐values are shown.

To address the value of TFx at T_1_, as we had done for baseline, we initially considered two groups of patients according to whether we detected ctDNA or not. Contrary to what we had observed at baseline, patients positive for TFx had shorter PFS and OS (Log rank test, *P* = 0.0023 and *P* = 0.00028, respectively; Fig. 3A, Fig. S2A, Table S3). Next, we examined ICI benefit across the three groups of patients using TFx = 10% as the cut‐off. Patients with undetectable TFx at T_1_ had substantially longer PFS than the other two groups (Log rank test, *P* = 0.000017 and *P* = 0.12, undetectable vs. TFx ≥ 10% and TFx < 10%, respectively, Fig. 3B), whereas shorter PFS was observed in the TFx ≥ 10% compared to TFx < 10% (Log rank test, *P* = 0.004, Fig. 3B). Similar data were obtained when considering OS (Fig. S2B, Table S3). Interestingly, none of the patients with objective response (partial response) had detectable TFx at T_1_. When comparing patients who achieved DCB to those who did not, TFx was significantly higher in the latter group (Mann–Whitney Wilcoxon, *P* = 0.0047, Fig. 3C), and indeed none of the patients with a TFx ≥ 10% at T_1_ achieved DCB (Fig. 3D).

We then addressed the value of TFx dynamics between B and T_1_, therefore, we established three groups: patients with undetectable TFx at both time points, patients who had TFx at baseline but cleared at T_1_ and patients with detectable TFx at both time points. Of note, none of the patients who had undetectable TFx at B exhibited detectable TFx at T_1_. Patients with detectable TFx at T_1_ had diminished PFS (and OS) compared to the two groups of patients with TFx undetectable at T_1_, (B detectable and T1 detectable vs. B detectable and T_1_ undetectable, and B detectable and T_1_ detectable vs. B undetectable and T_1_ undetectable, Log rank test, *P* = 0.032 and *P* = 0.0083, respectively, Fig. 3E,F). Interestingly, PFS was similar for those patients with undetectable TFx at T_1_ regardless of their TFx at baseline; OS followed a similar trend (Log rank test, *P* = 0.92 and *P* = 0.89, PFS and OS, respectively, Fig. 3E,F). These data indicate that TFx at T_1_ may reflect ICI benefit more accurately than baseline levels. Similar results were obtained when the groups were established based on the 10% cut‐off (Fig. S2C, Table S3).

It is worth highlighting that at T_1_, there was no association between TFx and the metastasis site, as it was observed at baseline (Fig. 2A, Fig. S2D). In fact, for few patients with liver metastasis (all had detectable TFx at B) we did not detect ctDNA at T_1_.

When considering two other time points, T_2_ and T_3,_ we obtained similar results to those obtained for T_1_ (Fig. S2E,F, Table S3), although the number of patients with plasma samples was lower, since some patients had already progressed. Considering these data and because patients may display different dynamics of response to ICIs, we addressed the value of TFx determination for any of the surveillance time points. None of the patients, who had a TFx ≥ 10% at any time point, experienced DCB. In fact, this patient group had substantially shorter PFS and OS compared to those patients who did not present a TFx ≥ 10% at any of the time points assessed (Log rank test, *P* = 0.000012 and *P* = 0.000017, respectively, Fig. S3A,B, Table S3).

Collectively, these data indicate that the study of ctDNA, using sWGS at different on‐treatment time points (even at 2–3 weeks aTI), provides valuable information with regard to the patient's clinical benefit following ICI treatment, regardless of the patient's values at B.

Finally, as we had samples drawn at baseline and at progression, we examined possible changes in the levels of TFx between both time points as the result of the treatment. We observed that samples at progression (*n* = 27) had a trend toward higher TFx than those at baseline (*n* = 40), although the increase did not reach statistical significance (Mann–Whitney *U* test, *P* = 0.2, Fig. S3C). In fact, this trend was not observed when restricting the analysis to the 24 patients with paired samples from both timepoints (Wilcoxon Signed‐Ranked test, *P* = 0.73, Fig. S3D); this is likely because patients, who had not progressed (and who tend to have lower TFx (B)), were only included in the unpaired analysis.

### 3.4 High SCNA burden correlates with diminished ICIs benefit

We had previously shown that high SCNA burden (of those events encompassing arms and chromosomes, that is aneuploidies), determined by sWGS on tissue biopsy, is associated with diminished PFS in patients with NSCLC treated with ICIs [14]. We, therefore, assessed its value when determined on baseline plasma samples. As we had detected reliable ctDNA in only 19 patients, we assessed its value as a continuous variable, using a univariate cox model. Our data indicate that SCNA burden of those events encompassing arms and chromosomes negatively associated with ICI benefit is significant for OS (*P* = 0.03) and close to significant for PFS (*P* = 0.07).

## 4 Discussion

In this study, we performed sWGS on plasma samples to evaluate the value of two distinct molecular parameters—TFx and SCNA burden—as biomarkers of ICI benefit in metastatic NSCLC. Owing to the minimally invasive nature of liquid biopsy, biomarkers that can be assessed with this approach have a great potential, since they can be easily determined over the course of the treatment. Thus, we applied sWGS on samples obtained prior to ICI initiation and at several time points during treatment until disease progression.

Several studies have explored different analytes employing liquid biopsy as a tool to evaluate treatment efficacy. Specific to NSCLC and ICI benefit, most studies have utilized gene‐panel sequencing on cfDNA from plasma. For example, baseline blood TMB (bTMB) has been shown to associate with ICI benefit [15, 16, 17], although its determination requires relatively large gene‐panels and may require correction for clonal hematopoietic mutations [29, 30]. Others used gene‐panels to monitor ctDNA kinetics to assess ICI benefit in NSCLC [17, 18, 19, 20] and found that an early‐on reduction of ctDNA levels is associated with more favorable clinical outcome. In fact, what has emerged from these and other studies is the concept of molecular response, as a decrease in the detection of ctDNA (using different thresholds).

However, fewer studies have employed sWGS on cfDNA—particularly in the context of NSCLC or ICIs. This approach was used to study PI3K inhibitors and chemotherapy efficacy in squamous NSCLC [27] and also in a pan‐cancer cohort of patients to study ICI benefit [31]. In fact, to the best of our knowledge, our study is the first ever to assess the value of applying sWGS on cfDNA in a cohort of patients with metastatic NSCLC treated with ICIs.

Our results suggest that the value of TFx, determined by sWGS, at baseline and in on‐treatment samples may provide different information. While at baseline, TFx might have a prognostic role, its value in the case of on‐treatment samples might be predictive of treatment efficacy, a concept already suggested by Zhang et al. [32] in a pan‐cancer cohort of patients with advanced disease who were treated with checkpoint blockade. First, we found that at baseline only high levels of TFx (≥ 10%) correlated (negatively) with ICI clinical efficacy. Conversely, its determination in on‐treatment samples seems to reflect ICI efficacy more closely. In fact, our results indicate that regardless of the levels of TFx at baseline, early‐on treatment TFx values were associated with ICI benefit. For instance, none of the patients that presented a TFx ≥ 10% at any of the surveillance time points achieved DCB. Secondly, we found that at baseline, there is a positive correlation between TFx and certain metastatic lesions (bone and liver), but this association was lost when treatment was initiated, indicating that TFx on‐treatment would reflect treatment efficacy. These data align with previous observations using gene‐panel sequencing, as discussed by Zhang et al. [32]. Furthermore, when using sWGS, other investigators found a positive association between bone metastasis and TFx in prostate cancer [33] and TFx and liver metastases in breast cancer [28].

Thus, sWGS may indeed represent a cost‐effective approach (compared to more expensive gene‐panels) to monitor the disease course, allowing multiple serial‐extractions without extensively increasing the financial costs. Furthermore, it does not require previous knowledge of the tumor's genome, nor is it limited by the detection of few mutations such as small panels or ddPCR.

Importantly, we in NSCLC [3, 14] and others [34, 35, 36] in other tumor types, have described a negative correlation between SCNA burden, determined in tissue biopsy, and ICI benefit. As sWGS on cfDNA enables us to estimate the SCNA burden, we have assessed its value as a biomarker of ICI benefit, using plasma samples. Although we could only determine the SCNA burden in half of our cohort, our data indicate that the SCNA burden is negatively associated with ICI benefit, even when adjusting for different features in a multivariate model. Interestingly, a recent study in urothelial cancer, applying an alternative technique to identify chromosomal aberrations on plasma samples, also found a negative association between aneuploidy burden (alterations in the copy number of chromosome and arms) and pembrolizumab (anti‐PD1) benefit [37]. Taken overall, results obtained with tissue samples and cfDNA, point to the value of SCNA burden (aneuploidy) as a pan‐cancer indicator of diminished ICI benefit. Nevertheless, its value in other ICI‐based therapies, such as ICIs plus chemotherapy, remains unexplored and should be addressed in future studies. As there are no currently reliable biomarkers identified that would definitively point to the best therapy for a given patient, the value of SCNA burden as a specific biomarker in this respect could, in fact, differ between ICI‐based therapies. Thus, the search for reliable biomarkers to support such clinical decisions is essential.

It is fair to say that our study has several limitations. After appropriate quality controls and (blind) manual curation of the SCNA profiles to maximize reliability of the data, we detected ctDNA in ~ 50% of patients in our cohort. This finding aligns with the concept that sWGS is less sensitive than other techniques (gene‐panel, amplicon‐sequencing or ddPCR). Also, it is important to mention, that due to the nature of the approach taken (sWGS plus IchorCNA [23]), the TFx and the SCNA burden are interdependent, and thus a combined analysis of both of these parameters was not possible. From a clinical perspective, the patients included in this study received different anti‐PD(L)1, and some were treated with combinations of checkpoint inhibitors. Furthermore, owing to the enrollment period of our patients, PD‐L1 expression was not available for all the patients and so we could only evaluate positive versus negative levels. Nevertheless, when adjusting for PD‐L1 expression in a multivariate model, TFx retained its significance. Although it would be beyond the scope of this work, it would certainly be interesting to establish the value of combinatorial biomarkers and jointly assess TFx dynamics, SCNA burden, TMB and some immune‐related biomarkers in plasma samples. The basis for such an assessment would be the suggestion that SCNA burden and TMB are likely independent biomarkers of response—at least in the case of tumor tissue samples [3], and that combining some biomarkers appeared to effectively predict DCB [17]. Finally, our analysis was retrospective and was based on our previous findings of the value of SCNA burden as a biomarker of ICI benefit, as shown with tissue samples [14].

## 5 Conclusions

Employing sWGS on cfDNA, our analysis suggests that TFx and SCNA burden are potential biomarkers of ICI benefit. TFx determination in on‐treatment samples may be more informative of therapy efficacy, whereas its determination at baseline may have more prognostic value (i.e., for certain metastatic lesions). Considering this and owing to its cost‐effectiveness, sWGS of plasma samples may very well be a useful tool to complement other biomarkers, especially for monitoring the disease course. Moreover, our data confirm previous observations regarding the negative correlation between SCNA burden and ICI benefit. Further studies with larger cohorts and combinatorial biomarkers will potentially help us to establish its value in NSCLC.

## Conflict of interest

EF reports consulting fees or speaker's bureau: AMGEN, AstraZeneca, Bayer, Bristol‐Meyers Squibb, Daichi Sankyo, Eli Lilly, F Hoffmann‐La Roche, Glaxo Smith Kline, Janssen, Medical Trends, Medscape, the healthcare business of Merck KGaA, Darmstadt, Germany, Merck & Co., Kenilworth, NJ, Novartis, Peervoice, Peptomyc, Pfizer, Sanofi, Takeda, Touchtime Oncology. Board: Grifols, independent member. Research funding: Fundación Merck Salud, Madrid, Spain; Grant for Oncology Innovation‐the healthcare business of Merck KGaA, Darmstadt, Germany to Vall d'Hebron Institut of Oncology. CC and JF: partial support through the Grant for Oncology Innovation, the healthcare business of Merck KGaA, Darmstadt, Germany. AVi reports consultant or advisory role: Guardant Health, Merck, Roche, Bristol‐Myers Squibb, Incyte, Bayer. Research funding: Bristol Myers Squibb, Roche, Incyte. Stock and Other Ownership Interests Reveal Genomics. EC reports Consultant or Advisory Role: AstraZeneca, Boehringer Ingelheim, Bristol‐Myers Squibb, Merck & Co., Kenilworth, NJ, Novartis, Roche, Takeda. Speaking: AstraZeneca, Boehringer Ingelheim, Bristol‐Myers Squibb, Merck & Co., Kenilworth, NJ, Novartis, Pfizer, Roche, Takeda. Grant support: Merk. Other: Bristol‐Myers Squibb, Pfizer, Roche, Takeda. TM declares consulting/advisory role fees from Roche, Bristol Myers, Boeringher, AstraZeneca, Lilly, and Research funding from Kyowa Kirin and Janssen, all of them unrelated with the present project. SC Consulting or Advisory Role: Bristol‐Myers Squibb Recipient, F. Hoffmann La Roche AG, Pfizer, Boehringer Ingelheim, Merck & Co., Kenilworth, NJ, Amphera. Travel Accommodations Expenses: F. Hoffmann La Roche AG, Pfizer, Boehringer Ingelheim. AC reports advisory role and/or travel compensation: Bristol‐Myers Squibb Recipient, F. Hoffmann La Roche AG, Pfizer, Boehringer Ingelheim, Merck & Co., Kenilworth, NJ, Kyowa Kirin, Celgene, Leo Pharma, Medscape, Kern Pharma. Astra Zeneca. PI reports advisory role and/or travel compensation: Bristol‐Myers Squibb Recipient, F. Hoffmann, La Roche AG, Merck & Co., Kenilworth, NJ, Boehringer Ingelheim, Merck & Co., Kenilworth, NJ, Rovi, Yowa Kirin, Grunenthal Pharma S.A., Pfizer, Medscape, Kern Pharma. AN reports advisory role, speaker's bureau or travel compensation: Bristol‐Myers Squibb, F. Hoffmann La Roche AG, Pfizer, Boehringer Ingelheim, Oryzon Genomics, Pfizer, AstraZeneca. AVa reports consulting or advisory role: Bristol‐Myers Squibb Recipient, F. Hoffmann La Roche AG, Pfizer, Boehringer Ingelheim, Merck & Co., Kenilworth, NJ, Amphera. Travel, Accommodations, Expenses: F. Hoffmann La Roche AG, Pfizer, Boehringer Ingelheim. NP reports advisory role and/or travel compensation: Merck & Co., Kenilworth, NJ, Bristol‐Myers Squibb Recipient, F. Hoffmann La Roche AG, Pfizer, Boehringer Ingelheim, Grunenthal Pharma S.A Kern Pharma. A.M.M. provided consultation, attended advisory boards and/or speaker's bureau for the following organizations: Bristol‐Myers Squibb, Lilly, F. Roche, Merck & Co., Kenilworth, NJ, Pfizer, Boehringer Ingelheim, AstraZeneca. All remaining authors declare no conflict of interest.

## Author contributions

Conception and design of the study: RA, JF, CC, and EF. Investigation: CC, JF, LL, RA, and EF. Acquisition of data: CC, LL, and NP. Resources: NP, AC, PI, AVi, MSa, AM‐M, AN, SC, TM, EC, MSo, AVa, and EF. Reviewed the clinical data: NP, AC, AV, PI, IP, EF, AM‐M, AN, SC, TM, and EC. Data curation: CC, AC, MSa, PI, NP, IP, MSo, and JG‐R. Formal analysis: JF. Project Administration: JG‐R, CC, and RA. Interpretation of the data: CC, JF, and RA. Writing—review and editing: All authors. Writing—original draft: RA. Funding acquisition: EF and RA.

### Peer review

The peer review history for this article is available at https://publons.com/publon/10.1002/1878‐0261.13409.

## Supporting information


**Fig. S1.** Overall survival results based on ctDNA detection at baseline.

**Fig. S2.** On‐treatment values of Tumor Fraction.

**Fig. S3.** On‐treatment values of Tumor Fraction and Tumor Fraction comparison between baseline and progression samples.

**Table S1.** Summary of clinical characteristics of the cohort.

**Table S2.** Detailed clinical characteristics of individual patients.

**Table S3.** Kaplan–Meier pairwise statistics for the different conditions related to supplementary figures.

**Table S4.** Multivariate Cox proportional‐hazards survival model (Progression‐free survival at baseline).

**Table S5.** Multivariate Cox proportional‐hazards survival model (Progression‐free survival at baseline).

**Table S6.** Multivariate Cox proportional‐hazards survival model (Progression‐free survival at baseline).

**Data S1.** Legends.

## Data Availability

The data that support the findings of this study are available on request from the corresponding authors. The data are not publicly available due to privacy or ethical restrictions.
