# Supplementary figures and images for: Dynamic changes in circulating tumor DNA assessed by shallow whole‐genome sequencing associate with clinical efficacy of checkpoint inhibitors in NSCLC
Source: Mol Oncol. 2023 Mar 21;17(5):779–91. doi: 10.1002/1878-0261.13409 (PMC10158763; doi:10.1002/1878-0261.13409)

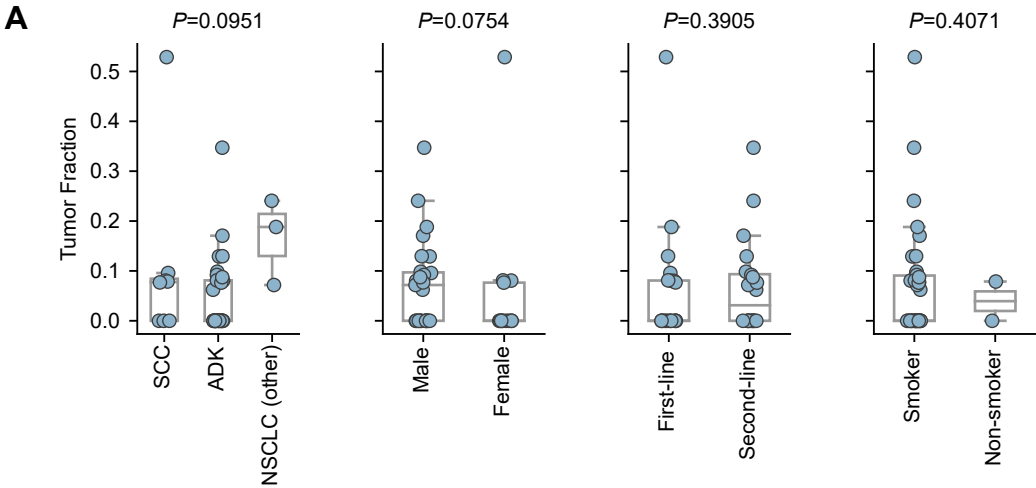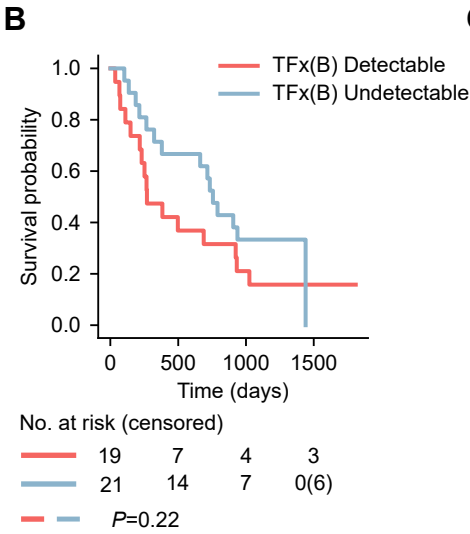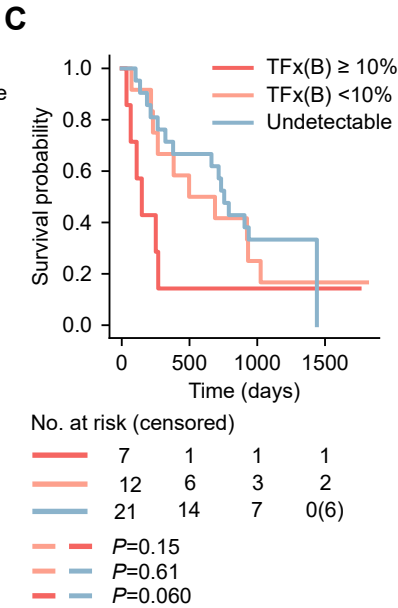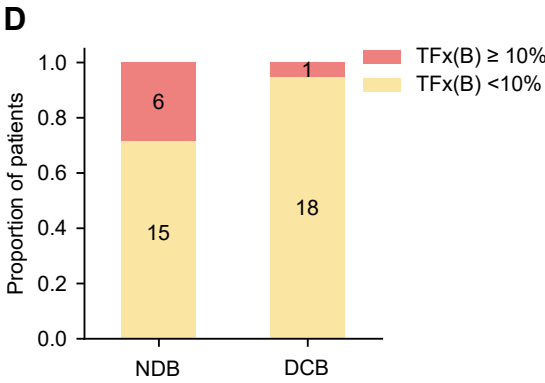

Supplement: Supplementary file 1 — Fig. S1. Overall survival results based on ctDNA detection at baseline. [file MOL2-17-779-s001.pdf]

**A**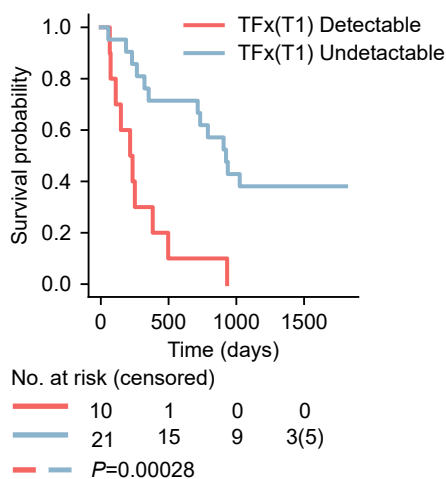**B**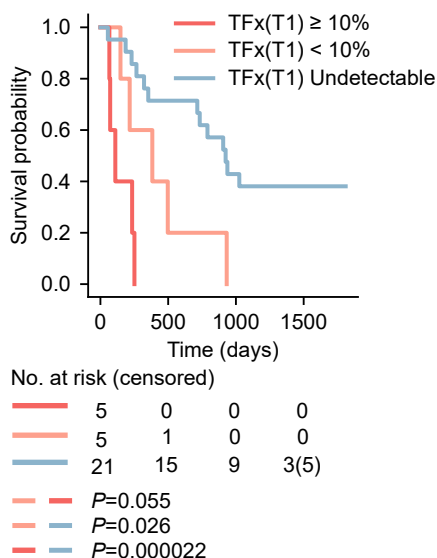**C**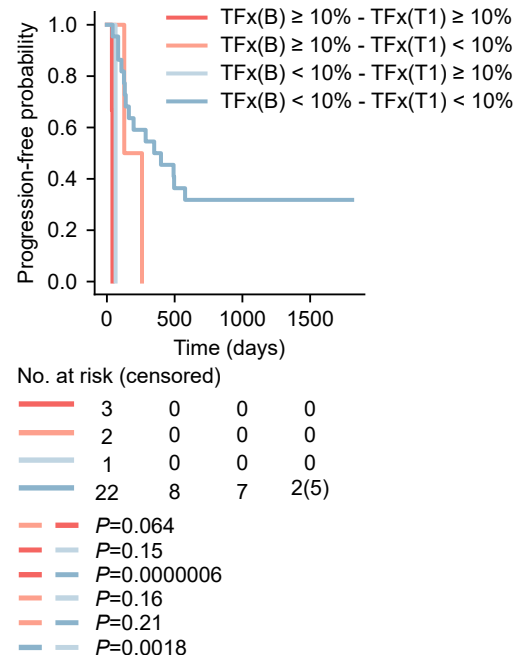**D**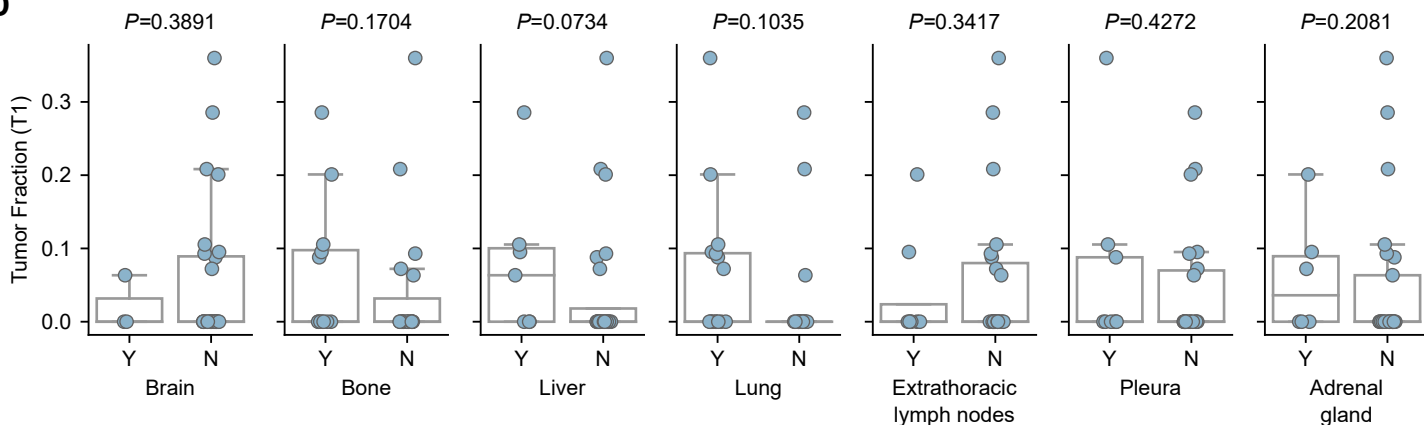**E**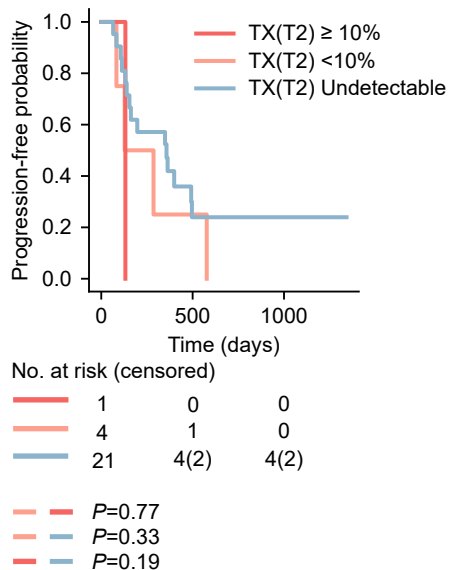**F**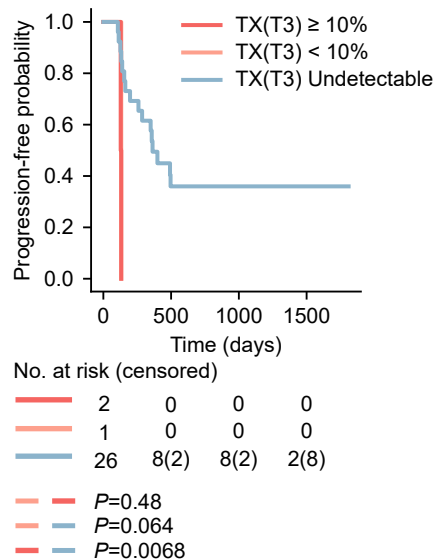

Supplement: Supplementary file 2 — Fig. S2. On‐treatment values of Tumor Fraction. [file MOL2-17-779-s007.pdf]

**A**

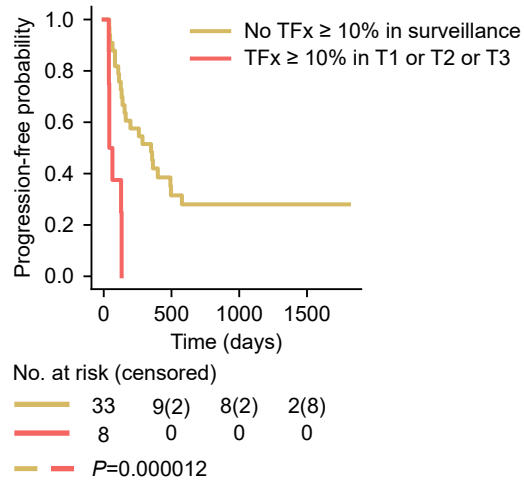

**B**

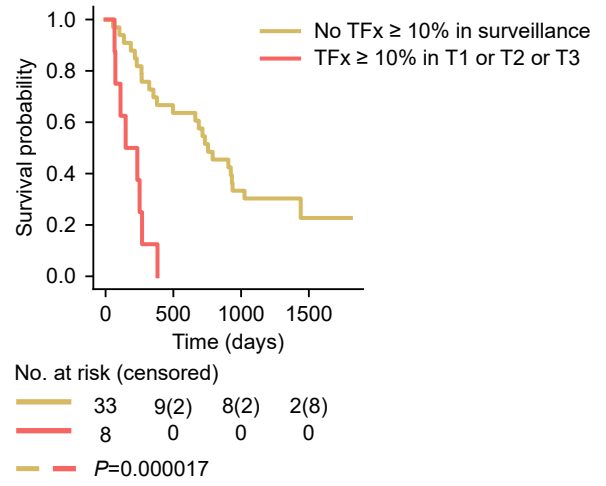

**C**

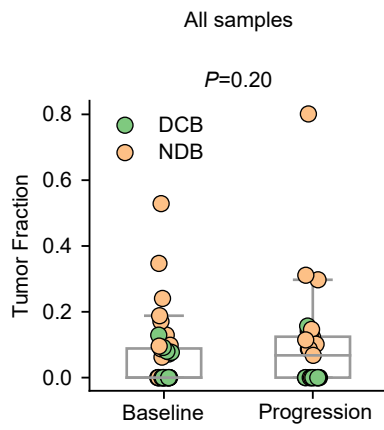

**D**

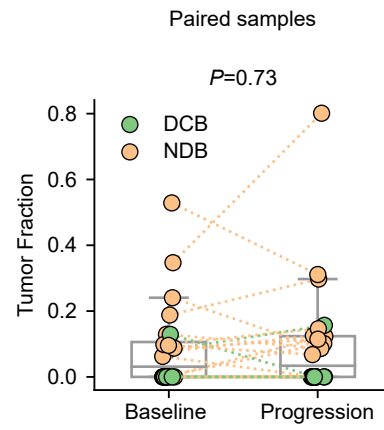

Supplement: Supplementary file 3 — Fig. S3. On‐treatment values of Tumor Fraction and Tumor Fraction comparison between baseline and progression samples. [file MOL2-17-779-s002.pdf]
