# Supplementary material for: Dynamic changes in circulating tumor DNA assessed by shallow whole‐genome sequencing associate with clinical efficacy of checkpoint inhibitors in NSCLC
Source: Mol Oncol. 2023 Mar 21;17(5):779–91. doi: 10.1002/1878-0261.13409 (PMC10158763; doi:10.1002/1878-0261.13409)
Supplement: Supplementary file 10 — Data S1. Legends. [file MOL2-17-779-s008.docx]

**SUPPORTING INFORMATION.**

**Supplementary Figure S1. Overall survival results based on ctDNA detection at baseline.** (A) Tumor fraction levels according to the category indicated. Mann–Whitney Wilcoxon, P-values are shown. The boxes show the quartiles of the dataset whereas the whiskers extend to show the rest of the distribution, except for points that are determined to be “outliers” by the seaborn library. (B) Kaplan-Meier survival curves of Overall survival (OS) in patients with undetectable ctDNA versus detectable ctDNA. Log rank test, P-value is shown. (C) OS in patients with undetectable ctDNA, patients whose Tumor Fraction (TFx) was <10% and patients whose TFx was ≥10%. Log rank test, P-values are shown. (D) Fraction of patients with TFx <10% and TFx ≥10% according to no durable benefit (NDB) versus durable clincal benefit (DCB).

**Supplementary Figure S2. On-treatment values of Tumor Fraction.** (A) Kaplan-Meier survival curves of overall survival (OS) in patients with undetectable ctDNA versus detectable ctDNA at T1. Log rank test, P-value is shown. (B) OS in patients with undetectable ctDNA, patients whose Tumor Fraction (TFx) was <10% and patients whose TFx was ≥10% at T1. Log rank test, P-values are shown. (C) Progression-free survival (PFS) in patients with TFx <10% at baseline and TFx <10% at T1 (TFx(B) < 10% - TFx(T1) <10%), TFx <10% at baseline and TFx ≥10% at T1 (TFx(B) <10% - TFx(T1) ≥10%), TFx ≥10% at Baseline and TFx <10% at T1 (TFx(B) ≥10% - TFx(T1) <10%) and TFx ≥10% at baseline and TFx ≥10% at T1 (TFx(B) ≥10% - TFx(T1) ≥10%). Log rank test, P-values are shown. (D) Tumor fraction levels according to the presence (Y) or absence (N) of the indicated metastatic lesion at T1. Mann–Whitney Wilcoxon, P-values are shown. The boxes show the quartiles of the dataset whereas the whiskers extend to show the rest of the distribution, except for points that are determined to be “outliers” by the seaborn library. (E, F) PFS in patients with undetectable ctDNA, patients whose Tumor Fraction (TFx) was <10% and patients whose TFx was ≥10% at T2 and T3, respectively. Log rank test, P-values are shown.

**Supplementary Figure S3. On-treatment values of Tumor Fraction and Tumor Fraction comparison between baseline and progression samples**. (A, B) Progression-free survival (PFS) and overall survival (OS) in patients with Tumor fraction (TFx) ≥10% at any time point (T1 or T2 or T3) versus patients who exhibited no TFx ≥10% at any of the time points. Log rank test, P-values are shown. (C) Tumor fraction at baseline or at progression considering all samples available or (D) in paired samples. Mann–Whitney Wilcoxon, P-values are shown. The boxes show the quartiles of the dataset whereas the whiskers extend to show the rest of the distribution, except for points that are determined to be “outliers” by the seaborn library. Durable clinical benefit (DCB), non-durable benefit (NDB).

**Supplementary Table S1. Summary of clinical characteristics of the cohort**.

**Supplementary Table S2. Detailed clinical characteristics of individual patients**.

**Supplementary Table S3. Kaplan-Meier pairwise statistics for the different conditions related to supplementary figures.**

**Supplementary Table S4 to S6. Multivariate Cox proportional-hazards survival model.**
